# Two coral fluorescent proteins of distinct colors for sharp visualization of cell-cycle progression

**DOI:** 10.1247/csf.23028

**Published:** 2023-06-30

**Authors:** Ryoko Ando, Asako Sakaue-Sawano, Keiko Shoda, Atsushi Miyawaki

**Affiliations:** 1 Laboratory for Cell Function Dynamics, RIKEN Center for Brain Science, 2-1 Hirosawa, Wako-city, Saitama 351-0198, Japan; 2 Department of Optical Biomedical Science, Institute for Life and Medical Sciences, Kyoto University, Kyoto 606-8507, Japan; 3 Biotechnological Optics Research Team, RIKEN Center for Advanced Photonics, 2-1 Hirosawa, Wako-city, Saitama 351-0198, Japan; 4 Laboratory of Bioresponse Analysis, Institute for Life and Medical Sciences, Kyoto University, Kyoto 606-8507, Japan

**Keywords:** fluorescent protein, cell cycle, time-lapse imaging, flow cytometry

## Abstract

We cloned and characterized two new coral fluorescent proteins: h2-3 and 1-41. h2-3 formed an obligate dimeric complex and exhibited bright green fluorescence. On the other hand, 1-41 formed a highly multimeric complex and exhibited dim red fluorescence. We engineered 1-41 into AzaleaB5, a practically useful red-emitting fluorescent protein for cellular labeling applications. We fused h2-3 and AzaleaB5 to the ubiquitination domains of human Geminin and Cdt1, respectively, to generate a new color variant of Fucci (Fluorescent Ubiquitination-based Cell-Cycle Indicator): Fucci5. We found Fucci5 provided more reliable nuclear labeling for monitoring cell-cycle progression than the 1^st^ and 2^nd^ generations that used mAG/mKO2 and mVenus/mCherry, respectively.

## Introduction

In recent years, there have been remarkable improvements in our ability to comprehensively unravel the fine details of cellular events. This is owing to the development of the green fluorescent protein from the jellyfish *Aequorea victoria* (avGFP), its spectral variants, such as cyan- and yellow-emitting variants (CFP and YFP, respectively), and GFP-like proteins including red-emitting fluorescent proteins (RFPs) from other organisms. These fluorescent proteins (FPs) can be incorporated into proteins by genetic fusion to develop genetically encoded probes for a variety of cellular functions (Rodriguez *et al.*, 2017).

**Fig. 1:**
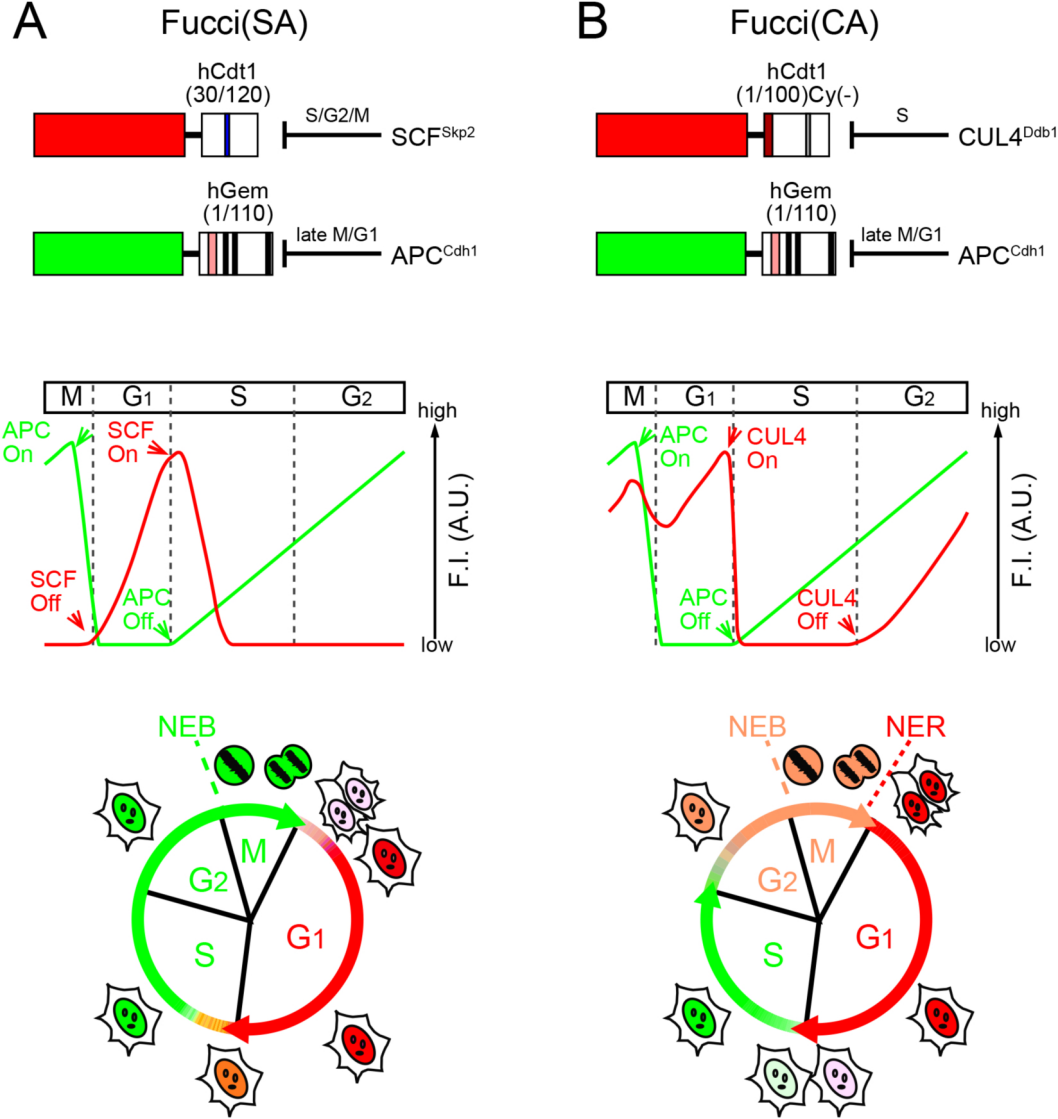
Fucci probes with different ubiquitination domains of human Cdt1 (A) Fucci(SA) consists of an SCF^Skp2^-sensitive hCdt1-based probe and an APC^Cdh1^-sensitive hGem-based probe. Fucci(SA) corresponds to the original Fucci. A blue box in hCdt1(30/120) indicates the Cy motif. (B) Fucci(CA) consists of a CUL4^Ddb1^-sensitive hCdt1-based probe and an APC^Cdh1^-sensitive hGem-based probe. The dark red box and the gray box in hCdt1(1/100)Cy(–) indicates the PIP box and Cy(–): mutated Cy motif , respectively. (A, B) Domain structures (top) and cell-cycle phasing capabilities (bottom) are shown, assuming that the hCdt1- and hGem-based domains are fused to red- and green-emitting FPs. A theoretical temporal profile of the fluorescence intensity (F.I.) is shown below each domain structure. SCF, SCF^Skp2^; CUL4, CUL4^Ddb1^; APC, APC^Cdh1^. Pink and black boxes in hGem(1/110) indicate the destruction box and nuclear localization signal, respectively. NEB: nuclear envelope breakdown. NER: re-formation of the nuclear envelope.

Fucci is an FP-based probe for visualizing cell-cycle progression (Sakaue-Sawano *et al.*, 2008). The technology harnesses the cell-cycle–dependent proteolysis of Cdt1 and Geminin (Fig. 1). Over the course of the cell cycle, SCF^Skp2^ and APC^Cdh1^ E3 ligase activities oscillate reciprocally and the protein levels of their direct substrates oscillate accordingly. Geminin, the inhibitor of Cdt1, is degraded under the control of APC^Cdh1^ E3 ligase. The original Fucci-S/G2/M probe had GFP or YFP fused to the APC^Cdh1^-mediated ubiquitination domain (1–110) of human Geminin (hGem(1/110)); this chimeric protein is the direct substrate of APC^Cdh1^ E3 ligase. On the other hand, the original Fucci-G1 probe had RFP fused to residues 30–120 of human Cdt1 (hCdt1(30/120)), which can serve as the direct substrate of SCF^Skp2^ E3 ligase. Thus, the original Fucci probe can be called Fucci(SA) because it monitors the balance between SCF^Skp2^ and APC^Cdh1^ E3 ligase activities. Fucci(SA) effectively highlights the transition process from G1 phase to S phase (Fig. 1A).

However, visualizing cell-cycle transitions other than G1/S is just as important. Thus, we next engineered the hCdt1-based RFP-containing probe to make it sensitive to CUL4^Ddb1^ instead of SCF^Skp2^. By combining the resultant probe with the hGem(1/110)-containing green or yellow probe sensitive to APC^Cdh1^, we developed Fucci(CA), which monitors the balance between CUL4^Ddb1^ and APC^Cdh1^ (Sakaue-Sawano *et al.*, 2017). Fucci(CA) distinguishes clearly interphase boundaries between G1, S, and G2 phases (Fig. 1B).

Fucci probes have been diversifying in color in the past decade. Initially, Fucci employed mAG (monomeric Azami Green) and mKO2 (monomeric Kusabira Orange2) (Sakaue-Sawano *et al.*, 2008). Then, the 2^nd^ generation, including Fucci(SA)2 and Fucci(CA)2, used mVenus and mCherry (Sakaue-Sawano *et al.*, 2011; Mort *et al.*, 2014: Sakaue-Sawano *et al.*, 2017). Also, far-red or near-infrared FPs have been substituted to generate Fucci variants for intravital deep imaging (Rodriguez *et al.*, 2016; Shcherbakova *et al.*, 2016). Very recently, lifetime-based Fucci has been developed using a pair of red-emitting FPs (Fucci-Red) (Shirmanova *et al.*, 2021) or a pair of newly-engineered HaloTag variants (*LT*-Fucci) (Frei *et al.*, 2022); these Fucci variants occupy only one spectral channel for cell-cycle monitoring and therefore are expected to contribute to optically multiplexed imaging. However, although Fucci technology has become a standard method for cell-cycle analysis in academia (Zielke and Edgar, 2015; Greenwald *et al.*, 2018; Eastman and Guo, 2020; Kohrman *et al.*, 2021), its spread to industry has been limited due to licensing restrictions of protein labeling techniques including FPs.

In the present study, we developed practically useful GFP and RFP named h2-3 and AzaleaB5, respectively, from corals, and substituted this GFP/RFP pair for the mVenus/mCherry pair in both Fucci(SA)2 and Fucci(CA)2 probes. The resultant probes: Fucci(SA)5 and Fucci(CA)5, respectively, would become more disseminatable than before.

## Materials and Methods

### cDNA cloning

The soft coral *Ricordia sp.* and the stony coral *Montipora monasteriata* were purchased from an aquarium shop. For each coral, whole tissue was frozen and ground down with a MultiBeads Shocker (Yasui Kikai), and total RNA was isolated by TRIzol Reagent (Thermo Fisher Scientific). mRNAs were purified using an Oligotex-dT30<Super> (JSR). cDNA was synthesized with a *Sal*I site at the 5' end and a *Not*I site at the 3' end by using a SuperScript^TM^ Plasmid System with Gateway^®^ Technology for cDNA Synthesis and Cloning (Thermo Fisher). Ligation of the cDNAs into a *Sal*I/*Not*I-cleaved pRSET-FastBac plasmid (Ando *et al.*, 2002) produced a directional cDNA library in a prokaryotic expression vector. The libraries were transformed into the *E. coli* strain JM109 (DE3). Colonies were screened for fluorescence by using a UV illuminator (365 nm) and a LED transilluminator (green).

### Mutagenesis

Site-directed and semi-random mutations were introduced according to our protocols as described previously (Sawano and Miyawaki, 2000). Error-prone mutagenesis was based on PCR using GoTaq DNA polymerase (Promega) supplemented with 1 mM MnCl_2_.

### Protein expression, spectroscopy, pH titration

The cDNA of the coding region of fluorescent proteins was amplified by using primers containing 5' *Bam*HI and 3' *Eco*RI sites. The restricted products were cloned in- frame into the *Bam*HI/*Eco*RI site of pRSET_B_ for bacterial expression. Proteins were expressed in *E. coli* and purified by Ni-NTA (QIAGEN). Then protein samples were desalted through a PD-10 column (GE Healthcare). *In vitro* spectroscopy was performed in 50 mM, HEPES-NaOH, pH 7.4. Absorbance spectra were acquired with a spectrophotometer (U-3310, Hitachi). Fluorescence measurements were performed using a microplate spectrophotometer (SynergyMx, BioTek). pH titration buffers used were below; 50 mM NaOAc-HOAc (pH 4.0–5.0), 50 mM KH_2_PO_4_-NaOH (pH 6.0), 50 mM HEPES-NaOH (pH 7.0–8.0), 50 mM Glycine-NaOH (pH 9.0–10.0), 50 mM Na_2_HPO_4_-NaOH (pH 11.0), 50 mM KCl-NaOH (pH 12.0). Molar extinction coefficients of FPs were calculated by the ratio of matured chromophore absorbance and denatured chromophore absorbance. This measurement was based on the fact that after alkali denaturation of these FPs, the chromophore containing a dehydrotyrosine residue conjugated to the imidazolone group absorbs light maximally at 447 nm with a molar extinction coefficient of 44,000 M^–1^ cm^–1^ (Shaner *et al.*, 2004). The fluorescence quantum yields were measured by an absolute PL quantum yield spectrometer (C9920-02, Hamamatsu photonics) in 50 mM HEPES-NaOH, pH 7.4.

### Pseudo-native gel electrophoresis

Purified proteins were mixed with 4× sample buffer (0.2 M Tris-HCl, pH 6.8, 8% SDS, 20% 2-mercaptoethanol, 40% glycerol, 0.4% BPB) and run on a 10% polyacrylamide gel without denaturation. The gel was imaged with a digital color CCD camera under UV irradiation.

### Gene construction

tFucci probes were constructed by concatenating the hCdt1-based probe, P2A sequence (Fang *et al.*, 2005; Kim *et al.*, 2011), and the hGem-based probe. We utilized a PiggyBac transposon system to generate cells that stably express tFucci probes (Aoki *et al.*, 2013). The mVenus-P2A-mCherry-hGem(1/110) gene in pPBbsr2 was used for the construction of tFucci(SA)5. DNA fragments encoding *Bam*HI-AzaleaB5-*Eco*RV-*Not*I-*Xho*I and *Xho*I-hCdt1(30/120)-P2A-*Eco*RI were amplified using primers, and digested products were substituted for the *Bam*HI-mVenus-P2A-*Eco*RI gene in mVenus-P2A-mCherry-hGem(1/110) in pPBbsr2 vector to produce AzaleaB5-hCdt1(30/120)-P2A-mCherry-hGem(1/110) in pPBbsr2. Then, DNA fragments encoding *Eco*RI-h2-3-*Not*I and *Not*I-hGem(1/110)-*Hpa*I were amplified using primers, and digested products were substituted for *Eco*RI-mCherry-hGem(1/110)-*Hpa*I gene in AzaleaB5-hCdt1(30/120)-P2A-mCherry-hGem(1/110) in pPBbsr2 vector. The final product AzaleaB5-hCdt1(30/120)-P2A-h2-3-hGem(1/110) was referred to as tFucci(SA)5 and the sequence has been deposited in the DDBJ/EMBL/GenBank database [LC334437]. Likewise, DNA fragments encoding *Bam*HI-AzaleaB5-*Xho*I and *Xho*I-hCdt1(1/100)Cy(–)-*Age*I were amplified using primers, and digested products were substituted for *Bam*HI-AzaleaB5-hCdt1(30/120)-*Age*I gene in AzaleaB5-hCdt1(30/120)-P2A-h2-3-hGem(1/110)in pPBbsr2 vector. The final product AzaleaB5-hCdt1(1/100)Cy(–)-P2A-h2-3-hGem(1/110) was referred to as tFucci(CA)5 and the sequence has been deposited in the DDBJ/EMBL/GenBank database [LC334438].

### Cell culture

HeLa cells (a subclone of HeLa.S3) were grown in Dulbecco’s modified Eagle’s medium (DMEM) (FUJIFILM Wako Pure Chemical Cooperation) supplemented with 10% fetal bovine serum (FBS) and penicillin/streptomycin. HeLa.S3 has been characterized to proliferate relatively fast with a doubling time of 15–18 hours (Sakaue-Sawano *et al.*, 2008).

### Establishment of stable cell lines

For the generation of HeLa cell lines stably expressing tFucci probes, the PiggyBac transposon system was employed (Aoki *et al.*, 2013). The pPBbsr-based tFucci probes and pCMV-mPBase (neo-) encoding the *piggyBac* transposase were co-transfected into HeLa cells using PEI (Polyethylenimine) at a ratio of 3:1. Transfected cells were selected with blasticidin S (InvivoGen) (50 μg/ml for 3 days and subsequently 10 μg/ml for 7–10 days). tFucci-expressing single cell clones were further isolated by limited dilution.

### Flow cytometry

Hoechst 33342 solution (56 μl of 1 mg/ml stock) (DOJINDO, Kumamoto, Japan) was added to a 10-cm dish containing HeLa/Fucci cells. After incubation for 30 min, cells were harvested and analyzed using a FACSAria II (BD Bioscience, San Jose, CA). h2-3 was excited by a 488-nm laser line (laser diode) and its emission was collected through 530/30BP; AzaleaB5 was excited by a 561-nm laser line and its emission was collected through 610/20 BP. Hoechst 33342 was excited by a UV Laser at 355 nm, and its emission was collected through 450/50 BP. The data were analyzed using FlowJo software (Tree Star). See Table II for details.

### Long-term time-lapse imaging

Cells were grown on 35-mm glass-bottom dishes in phenol red-free DMEM containing 10% FBS. Cells were subjected to long-term, time-lapse imaging using a computer-assisted fluorescence microscope (Olympus, LCV100) equipped with an objective lens (Olympus, UAPO 40×/340 N.A. = 0.90), a halogen lamp, a red LED (620 nm), a CMOS camera (Hamamatsu Photonics, ORCA-Flash4.0), differential interference contrast (DIC) optical components, and interference filters. The halogen lamp was used with BrightLine® single-band filter set (Semrock): “FITC-2024B” for observing the h2-3 fluorescence, and “mCherry-C” for observing the AxaleaB5 fluorescence. For DIC imaging, the red LED was used with a filter cube containing an analyzer. Image acquisition and analysis were performed using MetaMorph 6.37 and 7.10 software (Molecular Devices), respectively. See Table II for details.

### Widefield photobleaching

Live HeLa (S3) cells that expressed AzaleaB5 or AzaleaB5/I165M on 35-mm glass-bottom dishes were incubated in Hanks’ Balanced Salt Solution (HBSS) containing 15 mM HEPES-NaOH (pH 7.4) and imaged on an inverted microscope (IX-70, Olympus) equipped with a standard 75 W xenon arc lamp, a 40× objective lens (UPlanFLN 40×/1.3 NA), and a cooled CCD camera (CoolSNAP HQ2, Photometrics). The whole system was controlled with MetaMorph (Molecular Devices LLC., Sunnyvale, CA, USA). Cells were exposed to continuous unattenuated arc-lamp illumination through an Exciter: 550DF30 (Omega). At the same time, image acquisition was performed every 30 s with a short exposure time (50 ms) using a cube, which accommodated the following filters.

Exciter: 546DF10 (Omega) combined with an ND filter (10% transmittance)

Dichroic mirror: FF580-FDi01 (Semrock)

Emitter: FF01-641/75 (Semrock)

See Fig. S1.

### Manual cell tracking

Image processing was performed manually using the “Journal” functions implemented in MetaMorph (Molecular Devices). First, fluorescence images of AzaleaB5 and h2-3 were merged. In addition, DIC images acquired at slightly different focal planes were merged for delineating individual cell nuclei. This morphology observation was particularly useful for marking mitotic events. Time sequence data of tracked cells are saved in “TrackRef” files. The mean fluorescence intensities of tracked nuclei were calculated using the “Region measurements” function.

## Results and Discussion

**Fig. 2:**
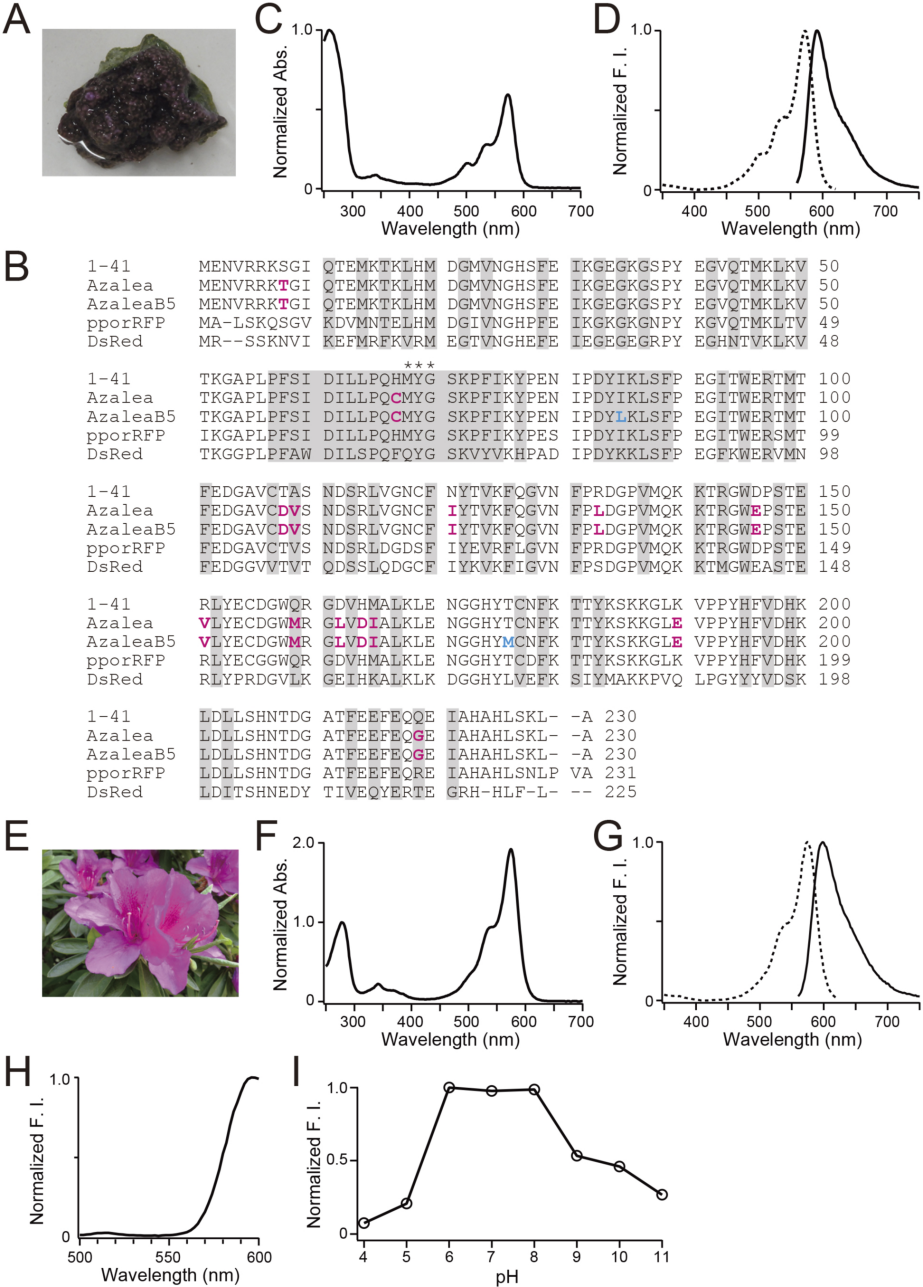
Molecular and spectroscopic characterizations of AzaleaB5 (A) *Montipora monasteriata*. (B) Amino acid sequence (single-letter code) alignments of 1-41, Azalea, AzaleaB5, pporRFP, and DsRed. Residues whose side chains form the interior of the β-barrel are shaded. Residues responsible for chromophore synthesis are indicated by asterisks. In the sequences of Azalea and AzaleaB5, the substituted amino acids in comparison with 1-41 are indicated in magenta. In the sequence of AzaleaB5, the substituted amino acids in comparison with Azalea are indicated in cyan. Many GFP-like proteins from Anthozoa form tetrameric complexes and have two interfaces: AB and AC. The AC interface has a large hydrophobic surface to be more stable than the AB interface. (C) Absorption spectrum of 1-41. The spectrum is normalized by the peak at 260 nm. (D) Normalized excitation (dotted line) and emission (solid line) spectra of 1-41. F.I., fluorescence intensity. (E) Azalea. (F) Absorption spectrum of AzaleaB5. The spectrum is normalized by the peak at 280 nm. (G) Normalized excitation (dotted line) and emission (solid line) spectra of AzaleaB5. F.I., fluorescence intensity. (H) Emission spectrum of AzaleaB5 with excitation at 480 nm. F.I., fluorescence intensity. (I) pH dependence of the fluorescence of AzaleaB5. F.I., fluorescence intensity.

**Fig. 3:**
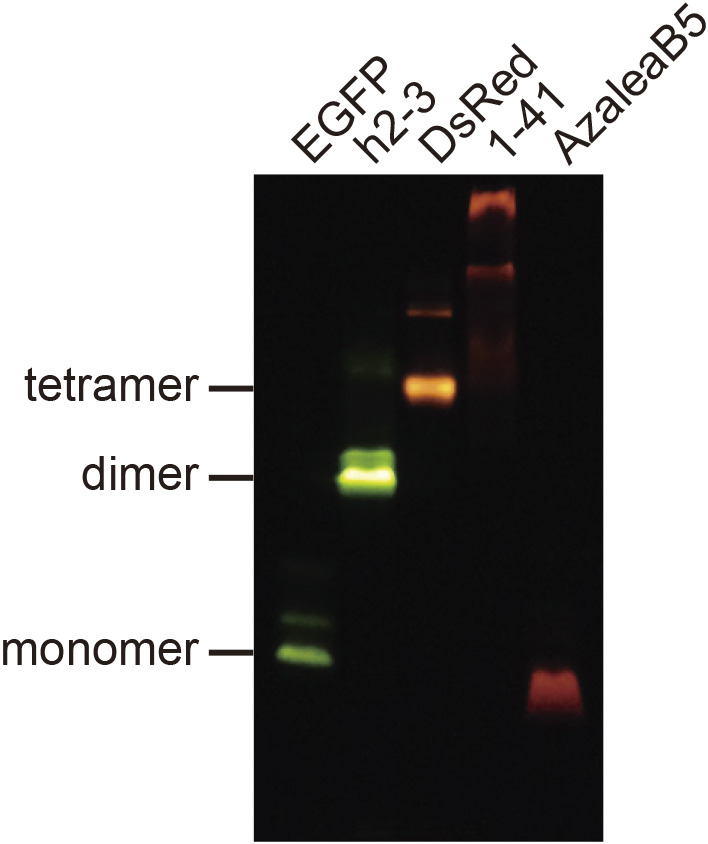
Pseudo-native gel electrophoresis analysis EGFP and DsRed were used as size markers (monomer and tetramer, respectively). The gel was illuminated with UV light (365 nm) and imaged using a color CCD camera.

We screened approximately 100,000 bacterial colonies containing a cDNA library prepared from *Montipora monasteriata* (Fig. 2A) for fluorescence. One clone was selected that appeared to encode an RFP, and temporarily referred to as 1-41. Based on an amino acid sequence alignment (Fig. 2B), 1-41 was supposed to have a similar β-can fold to other common FPs. The closest homologue was pporRFP, an RFP cloned from *Porites porites* (Poritiina, Poritidae) (Alieva *et al.*, 2008), which shared 80.8% identity. Transformation of the cDNA into *Escherichia coli* generated dim red fluorescent colonies. The addition of a histidine_6_ tag at the N-terminus of the protein allowed purification by metal affinity chromatography for spectroscopic and biochemical characterizations. The absorption spectrum of 1-41 at pH 7.4 displayed a major peak at 573 nm (Fig. 2C) and a slight shoulder at 537 nm; a small peak at 503 nm was indicative of a green-emitting byproduct (Miyawaki *et al.*, 2012). Excitation at around 540 nm produced weak fluorescence peaking at 592 nm (Fig. 2D). Pseudo-native gel electrophoresis analysis revealed that 1-41 formed a highly multimeric complex (Fig. 3).

We adopted semi-random mutagenesis to transform 1-41 into a useful RFP. We performed site-directed mutagenesis to break the multimeric structure, followed by random mutagenesis to rescue the red fluorescence (Campbell *et al.*, 2002; Ando *et al.*, 2004). We first introduced 14 mutations: S8T, H67C, T108D, A109V, N121I, R133L, D146E, R151V, Q159M, D162L, H164D, M165I, K190E, and Q219G into #1-41 (Fig. 2B). Among them, T108D was introduced into the AB interface, and D146E, R151V, D162L, and H164D were introduced into the AC interface. We found that M165I was effective in increasing the photostability of the red fluorescence (Fig. S1). The resultant RFP was practically bright and named “Azalea” after Wako City’s designated flower (Fig. 2E). Next, we used Azalea as the parental FP to develop several better mutants. One of them was AzaleaB5, which was generated by adding I85L and T176M into Azalea. Apparently, these two mutations further improved both brightness and folding efficiency. We also introduced silent base changes to optimize the coding sequence based on human codon-usage preferences. The absorption spectrum of AzaleaB5 at pH 7.4 displayed a major absorption maximum at 574 nm (ε = 104,000 M^–1^ · cm^–1^) with a slight shoulder around 542 nm (Fig. 2F). Excitation and emission spectra were analyzed to characterize the red-emitting component (Fig. 2G); the fluorescence quantum yield (QY) was 0.58. The spectral characteristics of AzaleaB5 are summarized in Table I. Excitation at 480 nm gave a negligible green emission compared with the red one (Fig. 2H), indicating that AzaleaB5 was free from contamination by the green-emitting component. The red fluorescence was stable at pH 6–8, but decreased with increasing acidity and alkalinity (Fig. 2I). Such alkaline sensitivity seemed to be unique to AzaleaB5; most conventional RFPs were stable in an alkaline as well as a neutral pH region. In pseudo-native gel electrophoresis, AzaleaB5 appeared to behave as a monomer (Fig. 3).

**Fig. 4:**
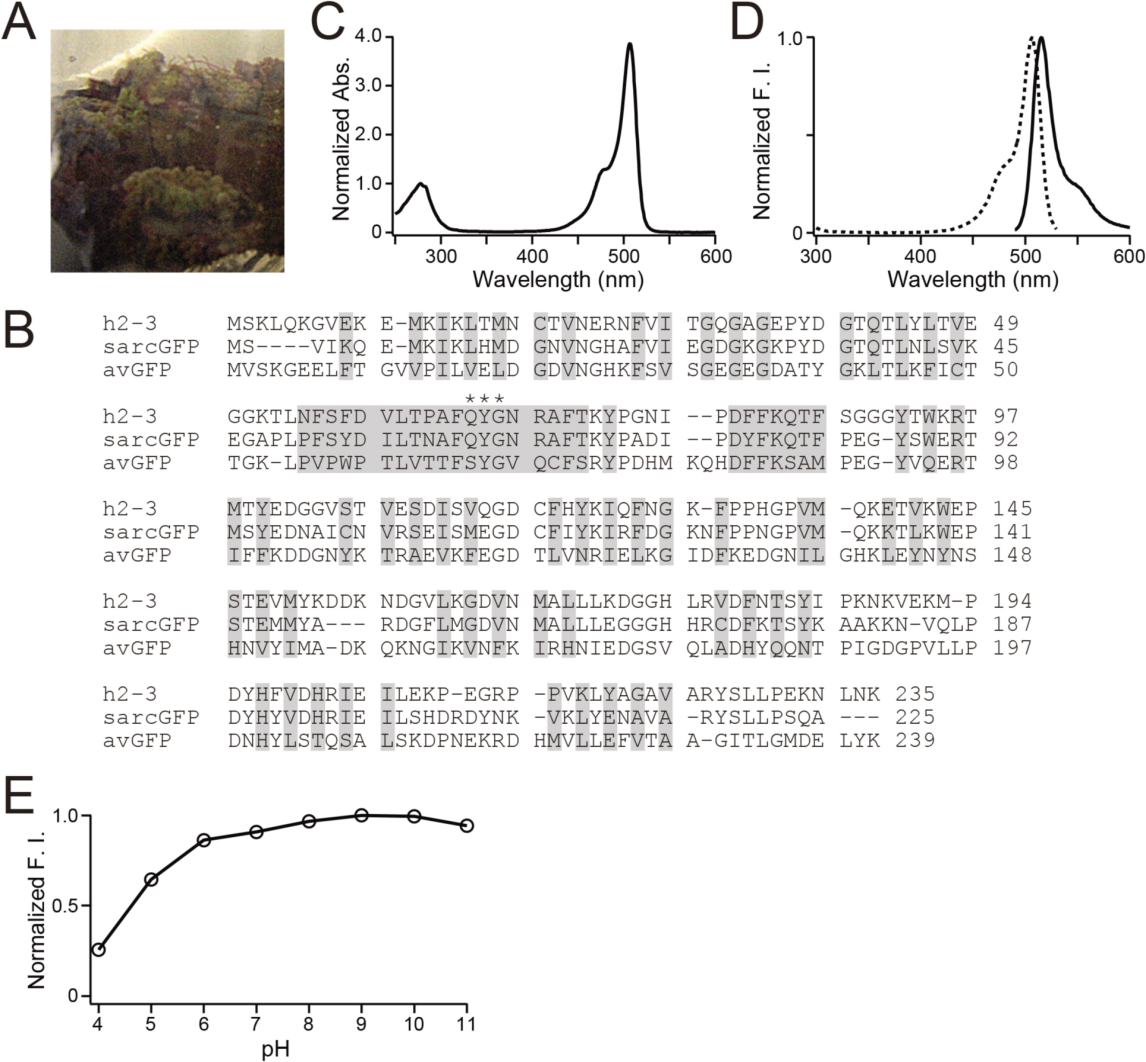
Molecular and spectroscopic characterizations of h2-3 (A) *Ricordea sp.* (B) Amino acid sequence (single-letter code) alignments of h2-3, sarcGFP, and *Aequorea victoria* GFP (avGFP). Residues whose side chains form the interior of the β-barrel are shaded. Residues responsible for chromophore synthesis are indicated by asterisks. (C) Absorption spectrum of h2-3. The spectrum is normalized by the peak at 280 nm. (D) Normalized excitation (dotted line) and emission (solid line) spectra of h2-3. F.I., fluorescence intensity. (E) pH dependence of the fluorescence of h2-3. F.I., fluorescence intensity.

We also cloned a cDNA that encoded a bright green-emitting FP from *Ricordea sp.* (Fig. 4A). The FP was temporarily referred to as 2-3. We also generated a mutated cDNA that encoded 2-3 with human codon-usage preferences. The resultant FP was named h2-3. Sequence analysis revealed that its nearest homologue was sarcGFP from *Sarcophyton sp.* (Octocorallia, Alcyoniidae) (Fig. 4B) (Alieva *et al.*, 2008). The absorption spectrum of h2-3 at pH 7.4 displayed a major absorption maximum at 506 nm (ε = 130,000 M^–1^ · cm^–1^) with a slight shoulder around 479 nm (Fig. 4C). The protein exhibited an emission spectrum peaking at 516 nm (Fig. 4D), which was sensitive to acidity with a p*K*_a_ of 4.6 (Fig. 4E). The spectral characteristics of h2-3 are summarized in Table I. It was shown by pseudo-native gel electrophoresis analysis that h2-3 formed an obligate dimeric complex (Fig. 3).

**Fig. 5:**
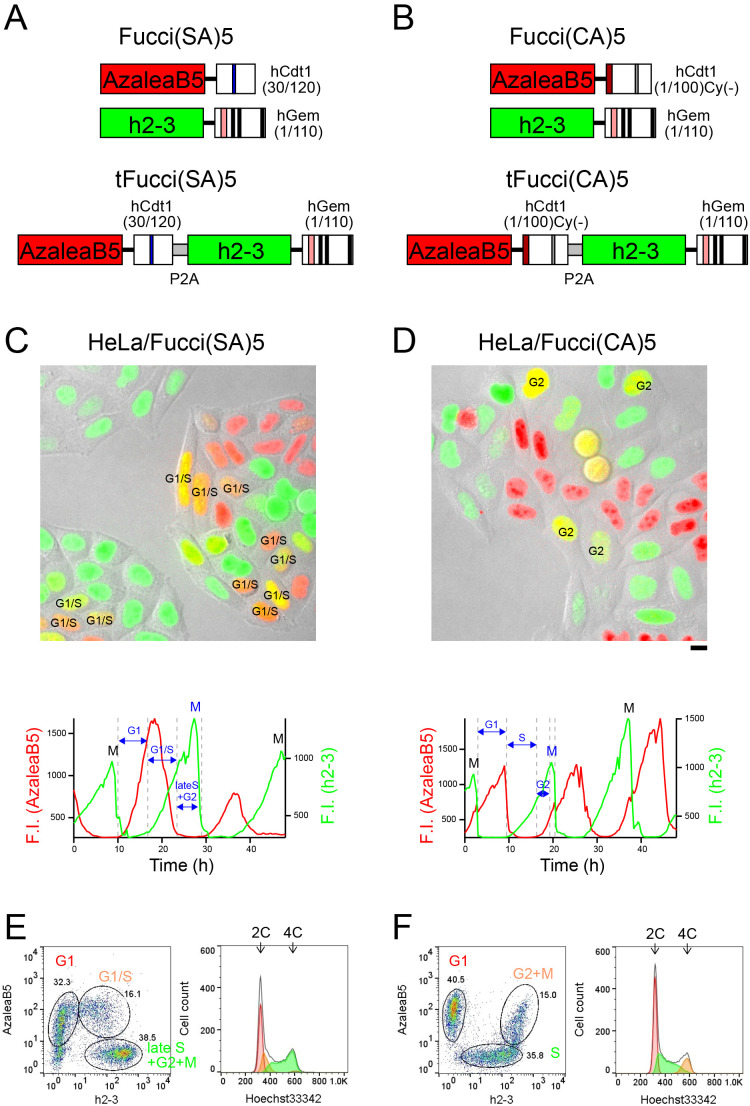
Characterization of Fucci(SA)5 and Fucci(CA)5 for cell-cycle progression in HeLa cells (A) Fucci(SA)5 and its tandem Fucci variant, tFucci(SA)5. (B) Fucci(CA)5 and its tandem Fucci variant, tFucci(CA)5. (C, D) Time-lapse imaging of HeLa/Fucci(SA)5 (C) and HeLa/Fucci(CA)5 (D). Fucci fluorescence and DIC images were merged. These cells were in the exponentially growing phase. Images were taken every 17 min and each experiment spanned 48 h. *top*, Snapshot images. Cells in G1/S transition (C) and cells in G2 phase (D) are highlighted. *bottom*, Temporal profiles of fluorescence intensities (F.I.) of AzaleaB5 and h2-3 are indicated by red and green lines, respectively. Interphase separation by Fucci probes are labeled in blue. M, mitosis. Scale bar, 10 μm. (E, F) Flow cytometry analyses of HeLa/Fucci(SA)5 (E) and HeLa/Fucci(CA)5 (F). *left*, Cells showing red [AzaleaB5(+)h2-3(–)], yellow [AzaleaB5(+)h2-3(+)], and green [AzaleaB5(–)h2-3(+)] fluorescence were gated for quantification of their DNA contents by staining with Hoechst 33342. *right*, C values denote DNA content as a multiple of the normal haploid genome.

Conventional Fucci2 (Sakaue-Sawano *et al.*, 2011; Mort *et al.*, 2014) is identical to Fucci(SA)2, which is composed of mCherry-hCdt1(30/120) and mVenus-hGem(1/110). On the other hand, Fucci(CA)2 is composed of mCherry-hCdt1(1/100)Cy(–) and mVenus-hGem(1/110). As Fucci(SA)2 and Fucci(CA)2 shared mVenus-hGem(1/110), we first manipulated this Geminin-based probe. Interestingly, hGem(1/110) can be fused to an FP that forms an obligate multimeric complex. For example, AmCyan, which forms an obligate tetramer, was successfully fused to hGem(1/110) to label S–G2–M-phase nuclei cyan (Nishimura *et al.*, 2013; Sakaue-Sawano *et al.*, 2017). Thus, we reasoned that the dimeric complex formation of h2-3 should not be a problem for fusion to hGem(1/110) to construct h2-3-hGem(1/110). Next, we constructed AzaleaB5-hCdt1(30/120) and AzaleaB5- hCdt1(1/100)Cy(–), and combined them with h2-3-hGem(1/110) to develop Fucci(SA)5 and Fucci(CA)5, respectively (Fig. 5A and 5B, top). The suffix “5” indicates that AzaleaB5 and h2-3 are used for fluorescence labeling. Furthermore, it was desirable that the Cdt1-based and Geminin-based probes be concatenated via the 2A peptide and encoded by a single transgene. This tFucci (tandem Fucci) approach guaranteed the stoichiometry of the two probes, thereby enhancing the reproducibility of cell-cycle analysis data obtained from cultured cells (Sakaue-Sawano *et al.*, 2017) and developing mouse embryos (Mort *et al.*, 2014). Accordingly, we constructed tFucci(SA)5 and tFucci(CA)5 (Fig. 5A and 5B, respectively, bottom), which were subcloned into pPBbsr2 vector. These plasmid DNAs were used to generate HeLa cells that stably expressed Fucci(SA)5 or Fucci(CA)5.

We examined the temporal profiles of the fluorescence intensities of AzaleaB5 and h2-3 by single-cell tracking analysis under a light microscope (Fig. 5C and 5D, Table II, Movie S1 and S2). We also investigated the cell-cycle monitoring behaviors of Fucci(SA)5 and Fucci(CA)5 by population analysis (Fig. 5E and 5F, respectively). After staining with Hoechst 33342 for 30 min, the cells were harvested and analyzed alive by flow cytometry (Table II). We noted that the cells labeled with yellow fluorescence by Fucci(SA)5 and Fucci(CA)5 had DNA contents of 2–4 C and 4 C, respectively.

**Fig. 6:**
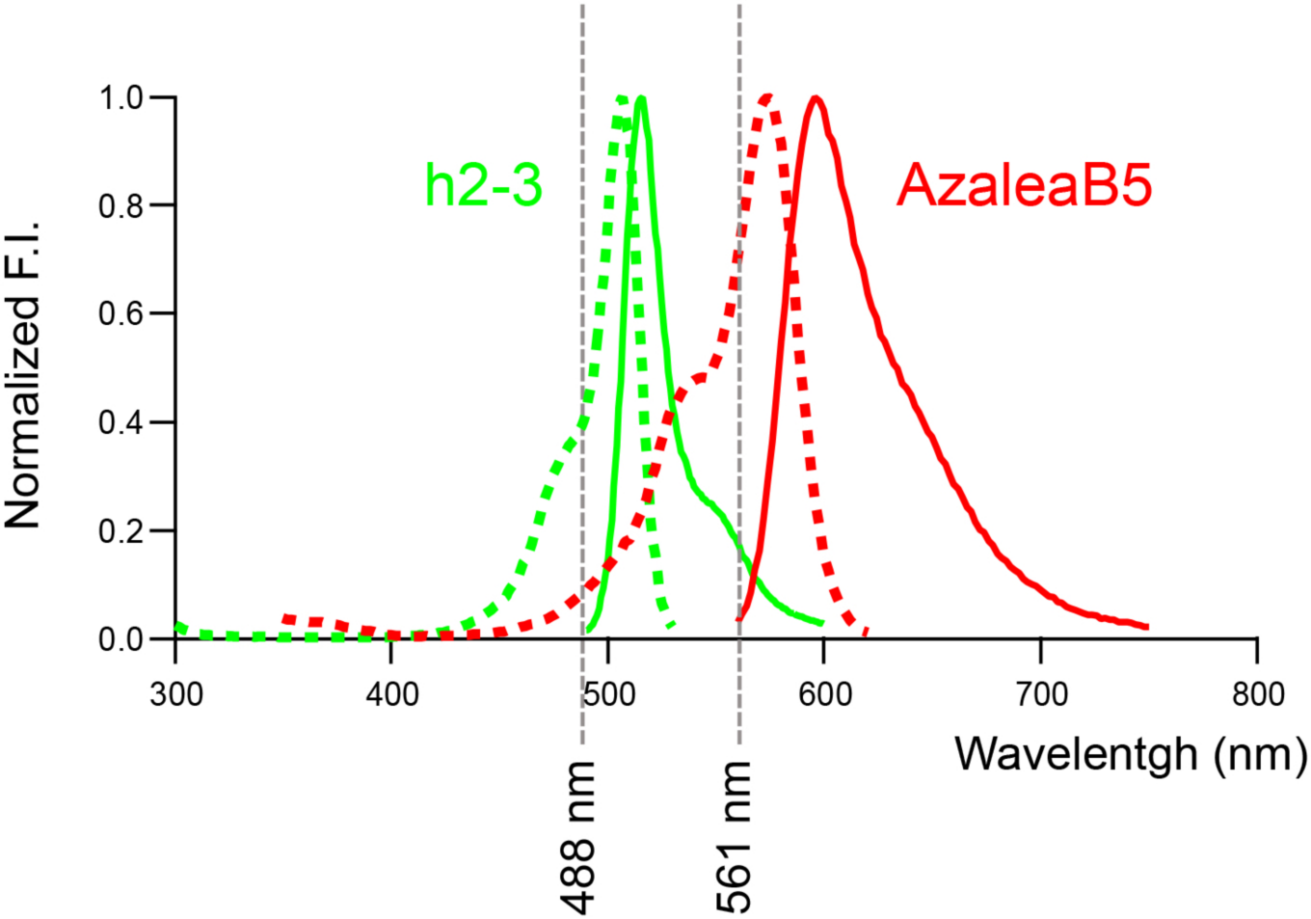
Spectral properties of AzaleaB5 and h2-3 with laser wavelengths Normalized excitation (dotted line) and emission (solid line) spectra of AzaleaB5 (red) and h2-3 (green) are shown.

The spectral properties of AzaleaB5 and h2-3 with the optical components of confocal fluorescence microscopy for dual-color imaging are shown in Fig. 6. These two FPs are excited best by common laser lines (488 and 561 nm) and their emissions are collected efficiently and specifically using a conventional multichroic mirror for the two laser lines. We have generated a human fibrosarcoma cell line (HT1080) that stably expressed tFucci(SA)5 or tFucci(CA)5. We confirmed that both HT1080/Fucci(SA)5 and HT1080/Fucci(CA)5 cells exhibited very bright nuclear labeling with green or red fluorescence. Likewise, we generated stable transformants of tFucci(SA)5 and tFucci(CA)5 using the human hepatocellular carcinoma cell line HepG2: HepG2/Fucci(SA)5 and HepG2/Fucci(CA)5, respectively. These Fucci5 cell lines will soon be used not only in the fields of basic life science and medical science but also extensively in the biotechnology industry, beginning with the drug discovery industry.

## Declaration of Interests

R. A. and A. M. are inventors on Japanese patent no. 6667897, US patent no. 10030055, EP patent no. 3037534 and Singapore patent no. 11201602289W that cover the creation and use of AzaleaB5. A. S.-S. and A. M. are inventors on Japanese patent no. 5370890, US patent no. 8182987 and EP patent no. 2138577 that cover the creation and use of Fucci.

## Data and Materials Availability

Genes: The AzaleaB5, h2-3, tFucci(SA)5, and tFucci(CA)5 genes are available from the RIKEN BioResource Research Center (BRC) at Tsukuba (http://en.brc.riken.jp/) under a material transfer agreement with RIKEN. The tFucci(SA)5 and tFucci(CA)5 in pPBbsr2 vector are available from addgene (https://www.addgene.org/153520/, https://www.addgene.org/153521/). The accession numbers in the DDBJ/EMBL/GenBank databases are [LC085679] for AzaleaB5, [LC085680] for h2-3 (under the name of FP2-3h), [LC334437] for tFucci(SA)5, and [LC334438] for tFucci(CA)5.

Stable cell lines: HeLa/Fucci(SA)5 (clone #16) (RCB 4917) and HeLa/Fucci(CA)5 (clone #16) (RCB 4919) cells are distributed by the RIKEN BioResource Research Center (BRC) Cell Bank (https://cell.brc.riken.jp/en/).

The information about Fucci-related materials is available in our website (https://cfds.riken.jp/material/fucci).

## Figures and Tables

**Table I:** Spectral properties of AzaleaB5 and h2-3

	Abs. max (nm)	Em. max (nm)	ε (10^3^ M^–1^ cm^–1^)	QY
AzaleaB5	574	596	104	0.58
h2-3	506	516	130	0.89

ε: molar extinction coefficient. QY: fluorescence quantum yield.

**Table II:** Optical components used for flow cytometry and time-lapse imaging

		DNA content	Fucci5	
		Hoechst/DAPI	h2-3	AzaleaB5
BD Biosciences FACSAria II	Excitation	355 nm	488 nm	561 nm
	Emission	450/50 BP	530/30 BP	610/20 BP
			“FITC-2024B”	“mCherry-C”
Olympus LCV100	Excitation		FF02-485/20	FF01-562/40
	Emission		FF01-524/24	FF02-641/75

## Supplementary Material

Movie S1 HeLa/Fucci(SA)5 cells were grown on a glass-bottom dish, and time-lapse imaging was performed using an LCV100 microscope. Images were acquired every 17 min. Total imaging time = 48 hr.

Movie S2 HeLa/Fucci(CA)5 cells were grown on a glass-bottom dish, and time-lapse imaging was performed using an LCV100 microscope. Images were acquired every 17 min. Total imaging time = 48 hr.
