# Supplementary material for: Two coral fluorescent proteins of distinct colors for sharp visualization of cell-cycle progression
Source: Cell Struct Funct. 2023 Jun 30;48(2):135–44. doi: 10.1247/csf.23028 (PMC10958192; doi:10.1247/csf.23028)
Supplement: Supplementary file 3 — Supplementary Materials [file csf_48_23028_3.zip › 48_23028_3/Legends to Movies S1 and S2.docx]

**Movie S1**

HeLa/Fucci(SA)5 cells were grown on a glass-bottom dish, and time-lapse imaging was performed using an LCV100 microscope. Images were acquired every 17 min. Total imaging time = 48 hr.

**Movie S2**

HeLa/Fucci(CA)5 cells were grown on a glass-bottom dish, and time-lapse imaging was performed using an LCV100 microscope. Images were acquired every 17 min. Total imaging time = 48 hr.
