# Supplementary material for: Two coral fluorescent proteins of distinct colors for sharp visualization of cell-cycle progression
Source: Cell Struct Funct. 2023 Jun 30;48(2):135–44. doi: 10.1247/csf.23028 (PMC10958192; doi:10.1247/csf.23028)
Supplement: Supplementary file 3 — Supplementary Materials [file csf_48_23028_3.zip › 48_23028_3/Supplementary Fig1.pdf]

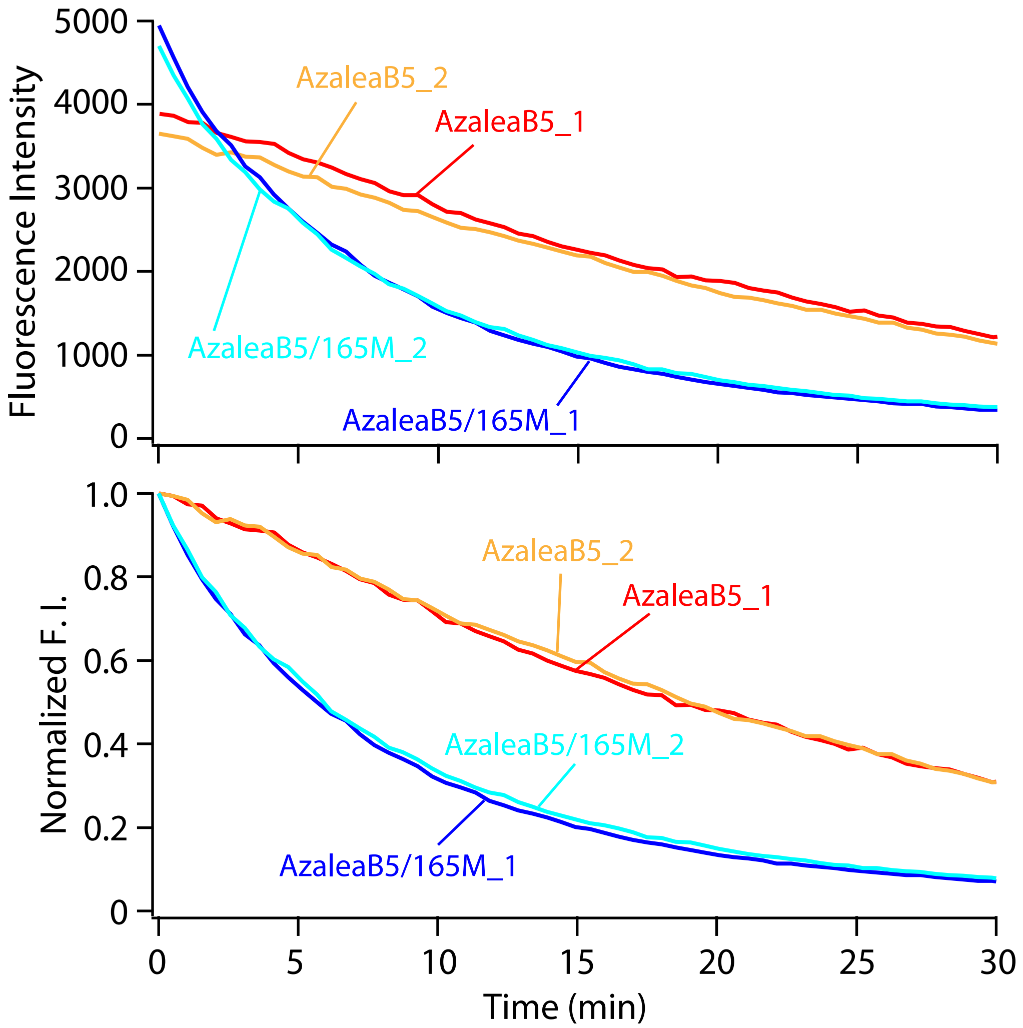


Fig. S1 Contribution of M165I mutation to photostability of AzaleaB5

With a back mutation of Ile165 to Met, AzaleaB5/I165M was generated. Photobleaching properties of AzaleaB5 and AzaleaB5/I165M were comparatively measured under continuous widefield illumination. Plotted as measured fluorescence intensity versus time (top) or as normalized fluorescence intensity versus time (bottom). For each construct, the average fluorescence intensity of three transfected cells in a field of view was measured (duplicated).
